# Decoding immune interactions of gut microbiota for understanding the mechanisms of diseases and treatment

**DOI:** 10.3389/fmicb.2023.1238822

**Published:** 2023-12-07

**Authors:** Qiang Yang, Ying Cai, Sifan Guo, Zhibo Wang, Yan Wang, Xiaodan Yu, Wanying Sun, Shi Qiu, Aihua Zhang

**Affiliations:** ^1^Good Agricultural Practices Center and Graduate School, Heilongjiang University of Chinese Medicine, Harbin, China; ^2^International Advanced Functional Omics Platform, Scientific Experiment Center, International Joint Research Center on Traditional Chinese and Modern Medicine, Hainan Engineering Research Center for Biological Sample Resources of Major Diseases (First Affiliated Hospital of Hainan Medical University), Key Laboratory of Tropical Cardiovascular Diseases Research of Hainan Province, Hainan Medical University, Haikou, China

**Keywords:** immune system, gut microbiota, metabolites, diagnosis, therapeutic strategies

Recently, several studies on gut microbes have been published in *Nature, Science*, and other series of articles to describe the relationship between gut microbes and body immunity (Bousbaine et al., 2022; Lyu et al., 2022; Mirji et al., 2022; Han et al., 2023; Tian et al., 2023). The above studies indicate that the gut microbiota can act as a vital immune organ to participate in the regulation of host immune homeostasis. Gut microbiota can promote the early development of the immune system, improve immune tolerance, and maintain normal communication between the immune system and the gut by secreting related metabolites (Figure 1). These studies illustrate the intrinsic relationship between gut microbiota and the immune system, which can be preferably used in diagnosis, prognosis, and treatment of tumors.

**Figure 1:**
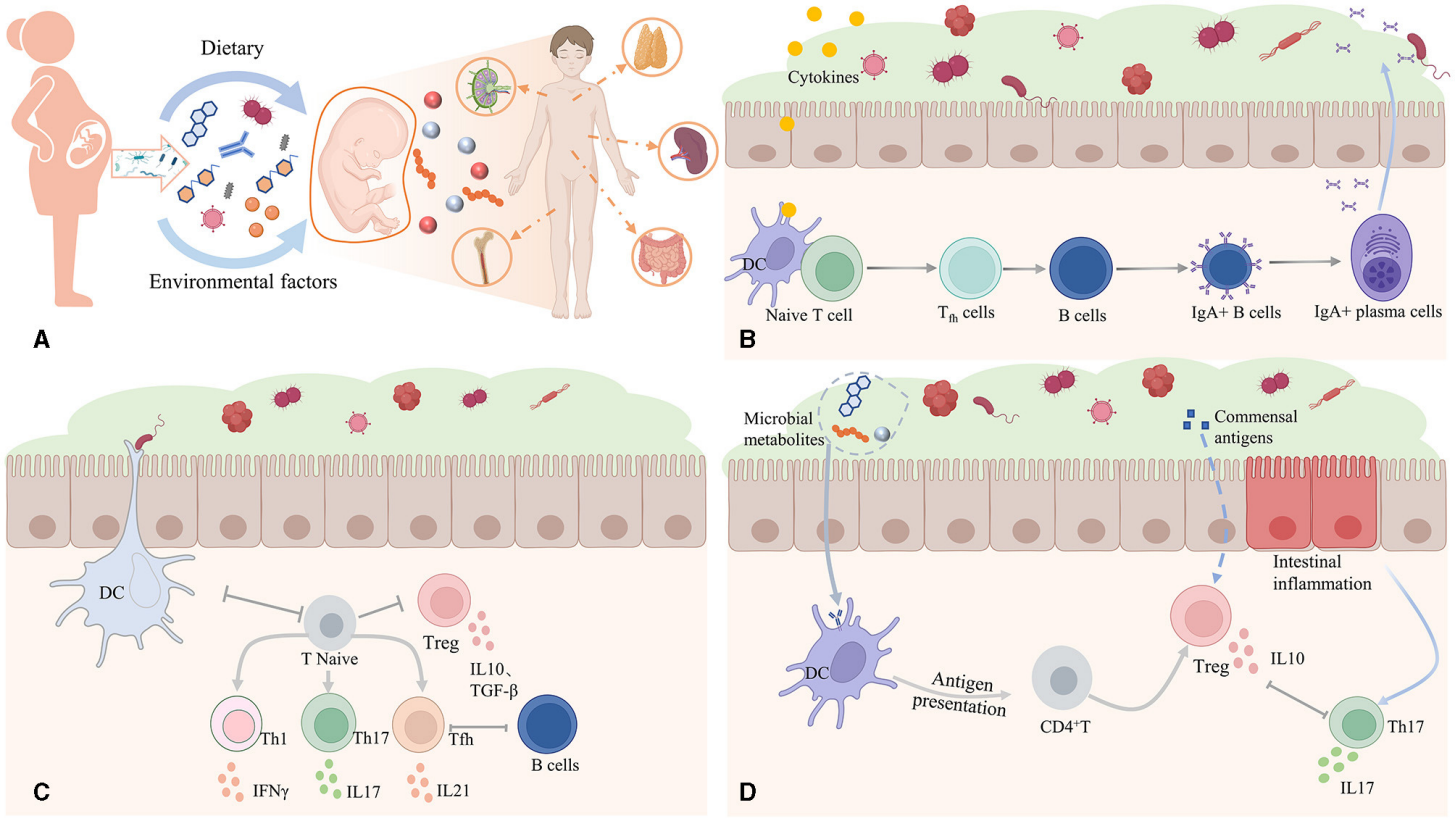
The intrinsic relationship between gut microbiota and the immune system. **(A)** Dietary and environmental factors can alter the gut microbiome, which can regulate and affect the metabolic, immune, and endocrine pathways and further affect fetal growth and development. **(B)** In response to microbial stimulation, host innate immune cells secrete inflammatory cytokines and chemokines. **(C)** Gut microbiota can target and regulate T lymphocyte differentiation and promote the improvement of immune self-tolerance mechanisms. **(D)** Gut microbes secrete various substances to build the gut barrier and contact the immune system. All images were obtained figures created by BioRender.

As the largest immune organ in the human body, the gut can promote the synthesis of vitamins, produce beneficial metabolites, fermented carbohydrates, promote intestinal peristalsis, regulate bile acids, compete with pathogenic bacteria, maintain the integrity of the intestinal barrier, and participate in the communication and shaping of the immune system (Qiu et al., 2023a). As an important immune participant, the gut microbiota maintains the stability of the human immune system in a variety of ways (Qiu et al., 2023b). By gaining further insight into the relationship between the microbiota and the immune system, it becomes possible to comprehend the microbiota's role in immune checkpoint blockades (ICBs) and other therapeutic strategies.

The gut microbiome sends information to the body about inchoate environmental exposures, such as diet and allergens, and promotes tolerance to them, helping the immune system recognize commensal bacteria and eliminate pathogenic bacteria. Numerous studies have shown that the primitive development of the immune system requires the cooperation of the gut microbiota, such as promoting the development of immune organs such as spleen and thymus, increasing the number of immune cells in lamina propria, and promoting the production of Immunoglobulin A (IgA) in intestinal mucus. Tian et al. (2023) found that antibiotic exposure in early life was associated with reduced gut microbiota diversity and abundance in adulthood. Hepatocyte interaction networks influenced by the gut-liver axis play a critical role in regulating Liver-resident NK (LrNK) maturation and function, which may be the key to the early development of the immune system. Early life is also a critical time for the interaction between gut microbiota and host immunity. Early life is a critical period for the development of intestinal flora and a critical window period for the maturation of the immune system. The development of the immune system requires the cooperation of intestinal microorganisms to maintain the homeostasis of gut microbiota and reduce susceptibility to a variety of immune diseases.

Gut microbiota can affect the function of almost all host organs and systems through a variety of signaling mechanisms. When innate immune cells in mucus come into contact with pathogenic bacteria, these cells secrete inflammatory cytokines and chemokines, recruit more innate immune cells, and may activate dendritic cells, and train the immune system to tolerate intestinal commensal bacteria and attack pathogenic bacteria. Continuous colonization of organisms by microbiota or pathogens can cause disorders of the immune system, which is dominated by immune cells. Lyu et al. (2022) found that immune cells of innate lymphoid cells (ILC3) play an important role in intestinal microbial tolerance, and the establishment and maintenance of immune tolerance is key to protecting host health.

Intestinal microbiota may regulate the differentiation of T lymphocytes into antigen-attacking effector T cells or antigen-tolerant regulatory T cells to promote the improvement of immune self-tolerance mechanisms. Bousbaine et al. (2022) found that β-hexosaminidase (β-hex) expressed by intestinal commensal bacteroidetes can be recognized by CD4 T cells as a conserved antigen of Bacteroidetes, thereby driving their differentiation into CD4+CD8αα+ intraepithelial lymphocytes. Some microbes in the gut modulate effector T cells in lamina propria by secreting peptides and cytokines, thereby releasing cytokines and inflammatory mediators in the affected intestinal regions.

The gut microbiota can secrete short-chain fatty acids, polysaccharides and produce other metabolites, which serve as key signaling factors and energy substrates to promote the establishment of the intestinal barrier, promote continuous communication between the gut and the immune system, and affect the development and metastasis of colon tumors. Intestinal microorganisms can regulate innate and adaptive immune responses, and there is a close relationship between metabolites derived from intestinal microorganisms and tumor immune response. The pro-inflammatory metabolite trimethylamine oxide (TMAO) was found to increase tumor immunoinfiltration and effector T cell activation, and enhance pancreatic ductal adenocarcinoma (PDAC) responsive to ICBs (Mirji et al., 2022). A comprehensive understanding of small molecule metabolites, molecular targets, and their biological significance produced by all gut microbes remains a pivotal orientation in the field. Han et al. (2023) found that indole-3-lactic acid (ILA), a metabolite of *Lactobacillus reuteri*, could exert anti-tumor effects by inhibiting the differentiation of IL-17 cells and inhibiting the transcriptional activity of the transcription factor RORγt. The above article further discusses intestinal flora metabolites as a complement to tumor and cancer prevention and treatment strategies, and analyzes the new relationship between intestinal flora metabolites and host immunity.

The interplay between gut microbiota and immunity is relatively obscure and complex. First, the gut microbiota can recognize nutrients and antigens by inducing the immune system to tolerate commensal bacteria. On the other hand, gut microbiota can also prevent bacterial invasion and infection through immune recognition. Modern studies have shown that gut microbiota and body immunity are interdependent. In some patients with autoimmune diseases, the diversity and abundance of gut microbiota are severely disrupted. Numerous other factors that may contract the gut microbiome, such as diet, medications, mental health, and environmental factors, are noteworthy for tumor or cancer treatment. Although we had a preliminary understanding of the gut microbiota, it is still unpredictable how the gut microbiota constitutes the anti-tumor immune response. The choice of a practical and viable treatment to change intestinal flora still needs to be made under the protection of an abundance of clinical trials. Prominently, methods for regulating and maintaining gut microbiota changes should be established in the context of preclinical models and clinical trials. By optimizing gut microbiota regulation and enhancing anti-tumor immunity and overall immunity, it is an opportunity to enhance immune surveillance and cancer therapy.

## Author contributions

QY, YC, SG, ZW, YW, XY, and WS participated in study design, contributed to method development, acquired funding, and wrote the manuscript. SQ and AZ advised on method development and contributed to manuscript generation. All authors contributed to the article and approved the submitted version.
